# Telemedicine as a Therapeutic Option in Sports Medicine: Results of a Nationwide Cross-Sectional Study among Physicians and Patients in Germany

**DOI:** 10.3390/ijerph18137110

**Published:** 2021-07-02

**Authors:** Stefan Hertling, Franziska Maria Loos, Isabel Graul

**Affiliations:** 1 Department of Obstetrics and Gynecology, University Hospital Jena, 07747 Jena, Germany; 2 Orthopedic Department, Campus Eisenberg, University Hospital Jena, 07607 Eisenberg, Germany; isabel.graul@icloud.com; 3 Practice for Orthopedics and Shoulder Surgery, 04177 Leipzig, Germany; loosfranziska@gmail.com; 4 Department of Trauma-, Hand- and Reconstructive Surgery, University of Jena, 07747 Jena, Germany

**Keywords:** eHealth, telemedicine, health services research, COVID-19, sports medicine, digitalization, telemedicine in sports medicine

## Abstract

Background: Worldwide, the number of treatments in the field of sports medicine is increasing. However, the COVID-19 pandemic has changed everyday life. Many consultations had to be cancelled, postponed, or converted to a virtual format. Telemedicine in sports medicine could support physicians. This study analyzes the use and perception of telemedicine applications among physicians and patients in the field of sports medicine in Germany. Methods: This prospective cross-sectional study was based on a survey of sports medicine physicians and patients in Germany during the COVID-19 pandemic. Descriptive statistics were calculated. Results: We analyzed the responses of 729 patients and 702 sports medicine physicians. Most believed that telemedicine is useful. Both physicians and patients rated their knowledge of telemedicine as unsatisfactory. The majority of respondents said they do not currently use telemedicine but would like to do so. Patients and physicians reported that their attitude had changed positively towards telemedicine and that their usage had increased due to COVID-19. The majority in both groups agreed on implementing virtual visits in stable disease conditions. Telemedicine was considered helpful for follow-up monitoring and prevention by both groups. Conclusion: Telemedicine in sports medicine has seen limited use but is highly accepted among physicians and patients alike. The absence of a structured framework is an obstacle to effective implementation. Training courses should be introduced to improve the limited knowledge regarding the use of telemedicine. More research in telemedicine in sports medicine is needed. This includes large-scale randomized controlled trials, economic analyses and explorations of user preferences.

## 1. Introduction

The global incidence of treating people in the field of sports medicine is increasing [1]. Different reasons are known: society is becoming older with increasing life expectancy; people are more health-conscious and have a healthier lifestyle; and in recent years, much has been studied in the area of health care and disease prevention. The central element is the healthy lifestyle. This includes a balanced diet and sufficient exercise [2]. As a result, more medical consultations are needed in the field of sports medicine [3]. Patients visit sports medicine physicians not only for the treatment of sports injuries themselves but also for advice on health issues and preventive health care [4]. Due to the COVID-19 pandemic, many medical treatments, especially in the field of sports medicine, have had to be postponed or cancelled. In addition to the changes in the acute treatment of the COVID-19 disease, many other changes have occurred in day-to-day medical care since then. However, the care of the patients has had to continue. New concepts and ideas have been considered.

The topic of digitization was driven forward by the COVID-19 pandemic. Digital media and applications can positively influence patient care and open up new treatment paths. Many physicians believe that telemedicine has great potential for managing patient care [5]. Patients are willing to use mobile health technologies to improve their disease status and to monitor symptoms and disease activity. The use of digital health applications has also increased in recent years [6].

The perspectives of patients and sports medicine physicians are crucial for the successful development and implementation of telemedicine concepts for the management of patient care in sports medicine. Telemedicine in sports can be used in for questionnaires and the above-mentioned health monitoring applications [7]. The central question is whether and how adequate treatment can be performed digitally in the future. This study explored the use and perception of digital health applications in the form of telemedicine applications by patients and sports medicine physicians undergoing treatment in Germany. Changes in these aspects were observed particularly during the COVID-19 pandemic.

## 2. Methods

Two surveys regarding the use of digital health applications in the form of telemedicine in the age of COVID-19 were administered to sports medicine physicians (specialists and trainees) and patients. The responsible ethics committee of the University in Jena was informed and did not object to the study (Reg.-No:2019-1456-Bef). These web-based surveys were conducted by members of the Working Group Young Forum of the German Society for orthopedics and trauma surgery (Arbeitsgemeinschaft Junges Forum der Deutschen Gesellschaft für Orthopädie und Unfallchirurgie (DGOU)). In order to investigate the identified areas of interest, a panel of experts conducted a questionnaire in two separate online meetings based on individual literature searches, similar to the EULAR-recommended standard operating procedures [8]. Four areas were investigated: (1) sociodemographic data, (2) the basic use of digital health applications, (3) knowledge and use of telemedicine and (4) the barriers and benefits of telemedicine in sports medicine. The study questionnaires have a web-based design according to published guidelines for questionnaire research [9,10,11]. The choice of questions for the questionnaire was based on both comparable work and on the quality criteria for online questionnaires [12]. The surveys were created in SurveyMonkey TM (SurveyMonkey, San Mateo, CA). The web-based surveys (SurveyMonkey Inc.) were conducted from 1 October 2020 to 30 April 2021. The data were collected anonymously. The studies were conducted in compliance with current data protection regulations and the Helsinki Declaration. The methodology and results were reported according to the checklist for reporting the results of Internet e-surveys [13]. Members of the Working Group Young Forum of the German Society for orthopedics and trauma surgery (DGOU) were asked to provide feedback on the format, completeness, clarity and procedure for the validation process [8,11]. Both surveys were pilot tested. The survey for physicians was administered to 10 sports medicine physicians and the patient survey was administered to 10 patients to gauge the need to refine wording and format and to check whether the predefined response options were exhaustive. Minor revisions were made. Accordingly, the questionnaire was modified. A 23-part, self-managed online questionnaire was developed for physicians and another for patients. They consisted of binominal questions, questions in categorical Likert scales (6 levels) and open questions and was entitled ‘Telemedicine as a Therapeutic Option in Sports Medicine’.

The main sections were as follows:

(a) the epidemiological data of respondents;
(b) the basic use of digital health applications;
(c) telemedicine: knowledge and use;
(d) telemedicine in sports medicine: barriers and benefits.

One aim of the survey was to shorten the interview duration to a maximum of 15 min in order to keep the dropout rate as low as possible and to motivate the respondents to answer as many questions as possible [14,15]. At the time of the survey, about 13,600 sports medicine physicians were working in Germany. Of these, almost 2300 worked in a hospital and 9200 of them worked in a private practice [16]. A total of 2993 sports medicine physicians worked in Central Germany. The physician survey was sent to 2993 sports medicine physicians in Central Germany (federal states of Thuringia, Saxony-Anhalt and Saxony). The contact details of potential participants in Central Germany were provided by the Association of Statutory Health Insurance Physicians [17]. The questionnaire was distributed to the physicians via e-mail. Participants were informed that their data would be strictly confidential and anonymous. Access to the study was granted with a survey link. Patients undergoing treatment in the special, “Sports Medicine” consultation hour had access to the online patient questionnaire via a QR code or survey link. The special consultation hour took place at Waldkliniken Eisenberg, a specialist orthopedic hospital. Waldkliniken Eisenberg is the largest university for orthopedics in Europe and the only one in Thuringia. It belongs to the University of Jena and enjoys an excellent reputation in the field of sports medicine.

All participants gave their consent. There were no exclusion criteria for participation. Only fully completed questionnaires were included in the subsequent analysis. The results were analyzed using Survey Monkey TM and the Statistical Package for the Social Sciences, SPSS (Version 22.0, SPSS Inc., Chicago, IL, USA). Descriptive statistics included quantities, percentages, median scores and ranges for ordinal variables. The chi square test was applied for the analyses of influencing parameters. The *p*-value of less than 0.05 was considered significant.

## 3. Results

### 3.1. Overview

The surveys were completed from October 2020 to April 2021. Of the 2993 physician questionnaires that were sent out, 732 (24.5%) were returned. Of the 732 responses, 30 (1.0%) were excluded from the analysis because fewer than half the questions were answered. The final response rate for sports medicine physicians was 23.5% (702/2993). In the period from December 2020 to April 2021, we treated 2340 patients in a special sports medicine consultation hour in our clinic. Of the 2340 patients, 789 participated in the study. Of the 789 responses, 60 (7.6%) were excluded from the analysis because fewer than half the questions were answered. The final response rate for patients was 31.2% (729/2340).

### 3.2. Epidemiological Data of Respondents

**Figure 1:**
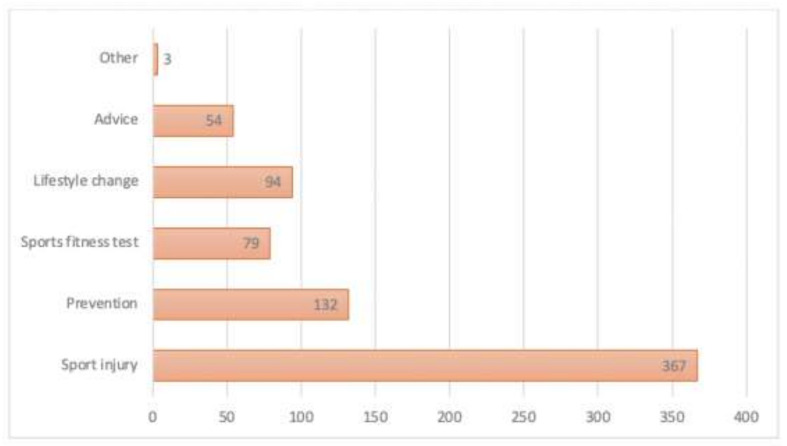
An overview of the individual treatment reasons of the patients.

Seven patients completed the survey. Most patients were between 31 and 40 years old. The majority of patients were male (*n* = 398, 54.6%). The majority of the participating patients were treated due to a sports injury. A total of 702 sports medicine physicians took part in the survey. Almost 83% were men (*n* = 583). Forty percent worked in a private practice, 32% (*n* = 225) were clinicians in a university hospital and 28% worked in a non-university hospital. Details of the participants are given in Table 1. An overview of the individual treatment reasons of the patients can be found in Figure 1.

### 3.3. Basic Use of Digital Health Applications (DHAs)

A percentage of 83.2% (*n* = 607) of patients reported using apps several times a day on a smartphone, 12.1% (*n* = 88) used apps once daily and 2.8% (*n* = 20) once weekly. Only 1.9% (*n* = 14) of the patients stated that they never used apps. Eighty-five percent (*n* = 617) of patients were able to use digital health applications. In addition, almost 75% (*n* = 547) said that the use of digital health applications can have a positive impact on their disease treatment, while almost 20% (*n* = 146) disagreed. The reason given by the patient for consulting a sports medicine physician showed no influence on the assessment of the value of digital health applications (*p* = 0.351). All physicians were able to use the digital health applications. Seventy-one percent (*n* = 498) of sports medicine physicians described the use of DHAs for managing the patient’s disease as useful and 10.2% (*n* = 72) disagreed. No significant difference in gender, age, or degree of training was noted. Physicians at university hospitals rated the usefulness of the digital health applications in sports medicine higher than physicians at non-university hospitals or practices (*p* < 0.001). Due to the COVID-19 pandemic, the attitude towards DHAs changed positively in 66.2% of patients (*n* = 483) and 52.8% of physicians (*n* = 371). Eighty percent of patients (*n* = 584) and 59.8% of physicians (*n* = 420) reported using DHAs more regularly (Table 2).

**Figure 2:**
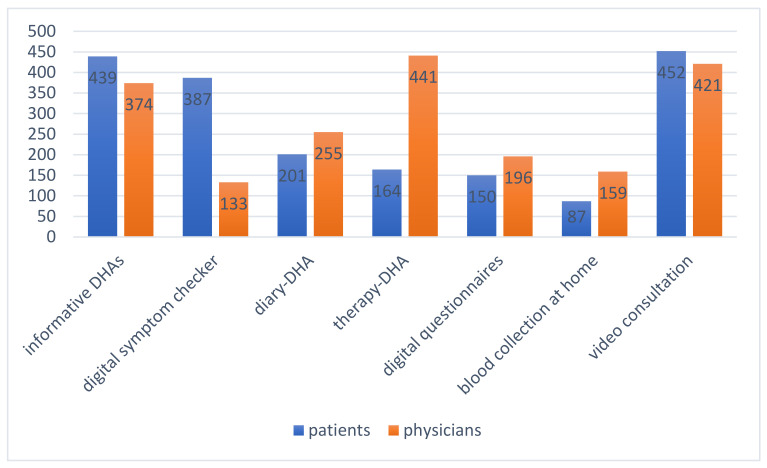
Attitudes towards the use of digital health applications among patients and physicians.

At the time of the survey, most patients said they were likely to use video consultations (62%, *n* = 452), informative DHAs (60.2%, *n* = 439) and symptom checkers (53.1%, *n* = 387). They stated that digital disease-related questionnaires and diary DHAs should be used more frequently in the future. Self-taken blood samples with digital access to the results showed different levels of acceptance: 88.1% of patients (*n* = 642) said they had no interest and 11.9% (*n* = 87) could imagine a future application of this technique. Most physicians stated that they were likely to use therapy DHAs (62.8%, *n* = 441), video consultations (60%, *n* = 421) and digital information DHAs (53.3%, *n* = 374). They stated that digital diary and digital-related questionnaires should be used more frequently in the future. Self-taken blood samples with digital access to the results showed different levels of acceptance: 77.4% of physicians (*n* = 543) said they had no interest and 22.6% (*n* = 159) could imagine a future application of this technique. The majority of physicians rejected the use of the symptom checker (79.8%, *n* = 569) (Figure 2).

**Figure 3:**
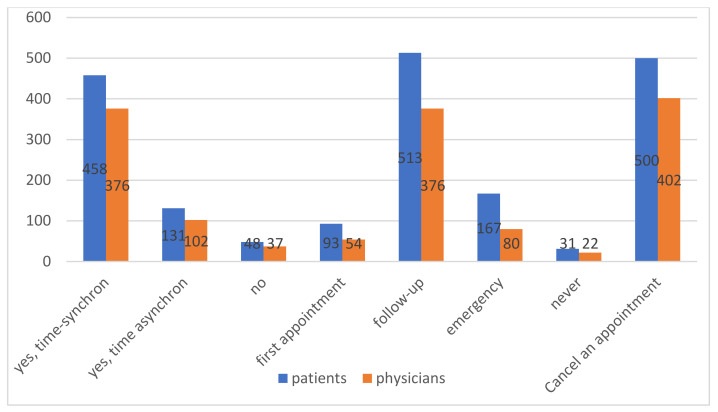
Attitudes towards video consultations among patients and physicians.

Patients stated that video consultations for aftercare (70.4%, *n* = 513) and emergency appointments (22.9%, *n* = 167) are possible. More than half (62.8%) (*n* = 458) of patients said that time-synchronous digital consultation could complement physical appointments. In addition, 68.6% (*n* = 500) of patients and 57.2% (*n* = 402) of physicians indicated that they should cancel an appointment on site if the patient’s disease is stable and can indicate well-being using a DHA (Figure 3).

### 3.4. Telemedicine from a Medical Point of View: Knowledge and Use

A total of 79.3% (*n* = 557) of physicians rated their knowledge of telemedicine as 4 (unsatisfactory), 5 (bad), or 6 (very poor). The minority (145/702, 20.7%) rated their knowledge of telemedicine as 1 (very good), 2 (good), or 3 (satisfactory). The majority (600/702, 85.6%) currently does not use telemedicine, but 69.5% (488/702) said they would like to use it. A total of 78.1% (548/702) of the surveyed physicians pointed out that they do not use telemedicine due to barriers. The three main obstacles to the introduction of telemedicine, according to the respondents, are the purchase of technology equipment (487/702, 69.3%), administration (430/702, 61.2%) and poor reimbursement (417/702, 59.4%) (Table 3).

### 3.5. Telesportsmedicine in Patient Care Management: Barriers and Benefits

A total of 84.2% (591/702) of the physicians considered telemedicine to be useful in sports medicine for patient care management. When asked who should interact with telemedicine, 78.1% (448/702) answered physician–physician, 61.1% (429/702) physician–patient and 26.7% (187/702) physician–assistant (multiple answers were possible). The preferred therapeutic phases for the use of telemedicine in the treatment of patients were follow-up (508/702, 72.3%), first contact (219/702, 31.2%) and preventive examinations (171/702, 24.4%). Participants were asked to provide specific digital tools that could support oncological care management for patients. The most frequently selected topics were teleconsulting (548/702, 78.1%), video consultations (463/702, 65.9%) and tele-diagnostics (407/702, 57.9%). This was followed by online appointments (273/702, 38.9%), e-learning (142/702, 20.2%), patient apps (100/702, 14.2%), digital screening (71/702, 10.1%), portable devices (70/702, 9.9%) and other instruments (29/702, 4.1%) (Table 4).

## 4. Discussion

This study was the largest nationwide survey on the use of telemedicine in Germany in the field of sports medicine for the promotion and implementation of telemedicine in the treatment of patients. For this purpose, patients and sports medicine physicians were interviewed. The results of a collaborative survey that evaluated the perspectives of patients and physicians during the COVID-19 pandemic were presented. The survey contained the following main topics: 1. epidemiological data of respondents; 2. basic use of digital health applications, 3. telemedicine: knowledge and use; 4. tele-sports medicine: barriers and benefits. In this survey study, patients and sports medicine physicians reported a positive attitude and increased the usage of DHAs due to the COVID-19 pandemic in Germany. In line with previous patient surveys [18], the majority of patients reported that they regularly used mobile apps on their smartphone and believed that they were able to use DHAs and that the use of DHAs may be beneficial for one’s own disease treatment. All physicians could use the digital health applications. This was the basis for the use of telemedical applications in the field of sports medicine. Physicians saw the overall use of telemedicine as acceptable and more than two thirds of respondents wanted to use telemedicine in their daily practice and would welcome the wide range of approaches to it. However, only a minority of physicians had already used telemedicine at the time of the survey. Barriers to the introduction of telemedicine in sports medicine, such as limited knowledge, high costs for the purchase of technical equipment and insufficient financial reimbursements, have been clearly identified. The results shed light on how telemedicine can support treatment in sports medicine from a medical and patient perspective. Familiar communication formats, such as the direct exchange of information with patients and medical colleagues, are leading in the field. Various tele-counseling tools have been developed, but this development is not as mature as it is in other disciplines, such as intensive care and cardiology. This is reflected in the small number of respondents who had used telemedicine at the time of the survey.

The online survey was designed to increase the response rate and to minimize efforts in data management. We aimed to obtain as high a return rate as possible by allowing respondents to complete the questionnaire within a short time, regardless of where and when they completed the survey. However, it can be assumed that this online survey is a positive distortion vis-à-vis users of telemedicine. To answer the questionnaire, knowledge of the field of telemedicine is required (e.g., preferences for specific tools were requested). Given the limited knowledge of physicians in the field of telemedicine, distortions are likely. In addition, we expect rapid technological developments in the field of telemedicine, so that the predefined response categories may not have been exhaustive enough. The survey was conducted in the time of COVID-19 and pre-pandemic data are pending in this area, so further research on the development of the acceptance of telemedicine applications in general and in relation to telemedicine in sports medicine is urgently needed. The average age of our sample corresponded to that of German physicians as a whole [19]. Men were slightly over-represented compared to the average [20]. This survey reflected only the opinion of sports medicine physicians. The survey was aimed at sports medicine physicians from Central Germany, especially physicians from Thuringia, Saxony and Saxony-Anhalt who participated in the recruitment strategy. We assume a self-selection bias and a nonresponse bias, because the survey was probably answered predominantly by physicians and patients interested in telemedicine.

This work provides basic knowledge regarding the application of telemedicine in the treatment of patients with sports injuries and an initial insight into the new field of telemedicine in sports medicine by providing detailed user settings, needs and barriers. We therefore believe that the results of this study in the development of new telemedicine solutions can help integrate these new solutions into the clinical routine of patients in sports medicine. In contrast to the results of a recent study, which revealed a negative attitude towards digitalization in the healthcare sector among physicians and patients in Germany [21], our results have shown that, among respondents, attitudes towards telemedicine have become more positive. A survey by the American Medical Association among nearly 3500 physicians in the United States found that less than 5% of sports medicine physicians used telemedicine, which is significantly fewer than physicians from other medical disciplines, such as radiologists (43%) [22] and less than the proportion of physicians using telemedicine according to our study. Although most respondents believe that tele-consultation can support the care of cancer patients, tele-consultation is rarely used. In a nationwide survey on digitization in the outpatient sector, respondents most commonly reported using e-mail only [23]. The main obstacles from the point of view of physicians are the security gaps in information technology (IT), the significant costs and the effort involved in the introduction of digital media technologies and an unfavorable cost–benefit ratio [24]. Respondents of our survey saw security vulnerabilities in IT as a rather minor problem. Digital consultations with patients appeared to have considerable potential in sports medicine, especially in follow-up visits [22]. However, only a minority of respondents were in favor of the use of telemedicine for initial consultations. This finding confirms the results of a comparable study from the United States of America [25]. In addition, the majority of physicians would prefer telemedicine to direct patient contact. This is comparable to the total telemedicine developments in the health sector [21,22]. Previous studies have shown that patients use telemedicine as a flexible solution that increases the independence of health authorities and personal knowledge [26]. Other studies suggest that health care created by video consultation sets is as effective as personal visits [27,28]. A qualitative study also reports that patients would be willing to accept electronic recordings and share patient reports (PROs) between clinical encounters when it is necessary to communicate with healthcare providers and access reliable information [28]. However, a recent study has shown that physicians hesitate to study electronic PROs because it would lead to a massive increase in their workload [29]. Mobile apps promise to speed up diagnostic examinations and improve monitoring [30]. The small number of sports medicine physicians who use apps to improve clinical routine contrasted with previous research from 2018, in which 49% said they already used such apps [31]. One of the main reasons for the reluctance to use apps may be the lack of proof [32]. Our results show that both patients and sports medicine physicians accept telemedicine. The age and gender of physicians showed no significant differences in telemedicine acceptance and preferences. Interestingly, physicians at university hospitals rated the value of DHAs significantly higher than physicians at other hospitals or practices. The reasons for this could be the lower cost pressure and the better availability of the required equipment. The more forward-looking attitude of the university hospitals could also represent a hurdle that is easier to overcome.

COVID-19 has highlighted the importance of non-contact approaches to medical care. In 2020, when the survey was conducted, patients and sports medicine physicians were willing to use telemedicine. It is assumed that this was a result of the pandemic. There has been an increase in the willingness to speed up the use of telemedicine as a social action, based on new standards in health care [23]. However, the great potential of telemedicine has not been fully achieved. Further research on implementation is urgently needed. These include large-scale randomized controlled studies on health effects, risks and incidents and specific interventions. Since our results show that there is no “one-size-fits-all” solution in the field of telemedicine, the perspectives and preferences of physicians, patients and other stakeholders in telemedicine in sports medicine are indispensable. This can create a basis for individual patient- and physician-adapted telemedicine options and triage mechanisms for the selection of patients for digital or analogue consultation [31,32]. Since physicians have reported on the barriers to the use of telemedicine, it seems that the structural framework for the effective implementation of telemedicine in sports medicine is not yet in place. A considerable administrative burden and inadequate reimbursement structures have prevented physicians interviewed from using telemedicine. The biggest obstacle, however, is the limited knowledge of physicians about the use of telemedicine, which is why it is necessary to provide information on telemedicine by introducing low-threshold training courses.

## 5. Conclusions

This study showed that patients and physicians support the implementation of telemedicine in sports medicine and two-thirds of those surveyed wanted telemedicine in their clinical routine. Those in the medical profession expressed an even greater willingness to use telemedicine. Respondents welcomed a variety of telemedicine approaches, but at present, only a minority of the physicians interviewed were using telemedicine. In addition, most physicians considered their knowledge of telemedicine to be rather poor. Further research and a reduction of existing barriers are urgently needed to improve high-quality telemedicine care, as well as training for specialists and generalists. Patients were very open to treatment with telemedicine applications. The foundations have been laid and development concepts in this area have great potential and should be explored. In addition, patients’ attitudes should be considered more intensively in the future.

## Figures and Tables

**Table 1:** Respondent characteristics (*n* (%)).

Sports Medicine Physicians (*n* = 702) (100%)	Patients (*n* = 729) (100%)
Men 583 (83)	Men 398 (54.6)
Age (years)	
21–30 169 (24)	153 (21)
31–40 204 (29)	284 (39)
41–50 147 (21)	139 (19)
51–60 98 (14)	88 (12)
>60 84 (12)	65 (9)
Consultant 498 (71)	
Resident 204 (29)	
Working place	
Private practice 281 (40)	
University hospital 224 (32)	
Non-university hospital 197 (28)	

**Table 2:** Use of digital health applications before and after the COVID-19 pandemic, *n* (%).

Characteristics	Patients (*n* = 729) (100%)	Physicians (*n* = 702) (100%)
I believe using digital health applications (e.g., medical apps, video consultation and online pharmacies) is useful for managing (my) disease, *n* (%)		
Strongly disagree	23 (3.1)	35 (5.0)
Disagree	37 (5.1)	37 (5.2)
Neutral	123 (16.8)	132 (18.8)
Agree	402 (55.2)	336 (47.9)
Strongly agree	144 (19.8)	162 (23.1)
Has your attitude towards digital health apps changed due to the COVID-19 pandemic?, *n* (%)		
It changed positively	483 (66.2)	371 (52.8)
It changed negatively	45 (6.2)	153 (21.8)
It has been unaffected	201 (27.6)	178 (25.4)
Do you use digital health apps more regularly since the COVID-19 pandemic?, *n* (%)		
Yes	584 (80)	420 (59.8)
No	145 (20)	282 (40.2)
I feel able to use digital health apps, *n* (%)		
Strongly disagree	8 (1.2)	0 (0)
Disagree	27 (3.9)	0 (0)
Neutral	77 (10.9)	0 (0)
Agree	520 (71.3)	506 (72.1)
Strongly agree	97 (13.8)	196 (27.9)

**Table 3:** Telemedicine: knowledge and use.

Question	Physician Responses: *n* (%)
How do you rate your own knowledge of telemedicine?	
total	702 (100)
1 (very good)	43 (6.2)
2 (good)	41 (5.8)
3 (satisfactory)	61 (8.7)
4 (unsatisfactory)	191 (27.2)
5 (poor)	210 (29.9)
6 (very poor)	156 (22.2)
Do you use telemedicine?	
total	702 (100)
yes	102 (14.4)
no	600 (85.6)
Would you like to use telemedicine?	
total	702 (100)
yes	488 (69.5)
no	214 (30.5)
Does anything prevent you from using telemedicine?	
total	702 (100)
yes	548 (78.1)
no	154 (21.9)
What prevents you from using telemedicine? (multiple selections possible)	
total	702 (100)
Purchase of technology equipment	487 (69.3)
Administration	430 (61.2)
Poor reimbursement	417 (59.4)
Data security	341 (48.6)
Lack of participation by colleagues	233 (33.2)
Technical comprehension of patients	156 (22.2)
Poor internet connection	82 (11.7)

**Table 4:** Implementation of telemedicine in sports medicine in patient care management.

Question	Physician Responses *n* (%)
Is telemedicine usable in sports medicine?	
total	702 (100)
yes	591 (84.2)
no	111 (15.8)
Which parties should establish communication via telemedicine? (multiple selections possible)	
Total	702 (100)
Physician–physician	448 (78.1)
Physician–patient	429 (61.1)
Physician–assistant	187 (26.7)
Other participants and combinations	93 (13.3)
No communication	48 (6.9)
At which stages can telemedicine support patient care management in sports medicine? (multiple selections possible)	
Total	702 (100)
Screening	171 (24.4)
Initial contact	219 (31.2)
Follow-up	508 (72.3)
Other stages	87 (12.4)
At no stage	46 (6.6)
Which tools could support patient care management in sports medicine? (multiple selections possible)	
total	702 (100)
Telecounseling	407 (57.9)
Telediagnostics	152 (37.9)
Video consultations	273 (65.9)
Online appointment assignments	273 (38.9)
e-Learning	142 (20.2)
Patient apps	100 (14.2)
Digital screening	71 (10.1)
Wearable devices	70 (9.9)
Other tools	29 (4.1)
No tools	10 (1.4)

## Data Availability

Data is contained within the article.
